# The Potential Role of Sulbactam and Cephalosporins Plus Daptomycin Against Daptomycin-Nonsusceptible VISA and H-VISA Isolates: An In Vitro Study

**DOI:** 10.3390/antibiotics8040184

**Published:** 2019-10-14

**Authors:** Chih-Cheng Lai, Chi-Chung Chen, Ying-Chen Lu, Tsuey-Pin Lin, Hung-Jui Chen, Bo-An Su, Chien-Ming Chao, Yin-Ching Chuang, Hung-Jen Tang

**Affiliations:** 1 Department of Internal Medicine, Kaohsiung Veterans General Hospital, Tainan Branch, Tainan 71051, Taiwan; dtmed141@gmail.com; 2 Department of Medical Research; Chi Mei Medical Center; Tainan 71004, Taiwan; ccomm2@yahoo.com.tw (C.-C.C.); chuangkenneth@hotmail.com (Y.-C.C.); 3 Department of Food Science, National Chiayi University, Chiayi 60004, Taiwan; biolyc2016@gmail.com; 4 Department of Health and Nutrition, Chia-Nan University of Pharmacy and Science, Tainan 71710, Taiwan; tplin007@mail.cnu.edu.tw; 5 Department of Medicine, Chi Mei Medical Center, Tainan 71004, Taiwan; uolddy@gmail.com (H.-J.C.); suboan0421@gmail.com (B.-A.S.); 6 Department of Intensive Care Medicine, Chi Mei Medical Center, Liouying, Tainan 73657, Taiwan; ccm870958@yahoo.com.tw

**Keywords:** vancomycin-intermediate resistant *S. aureus*, heterogeneous vancomycin-intermediate *S. aureus*, daptomycin, cephalosporin, sulbactam, synergism test

## Abstract

This study assesses the synergistic effect of the combination of cephalosporins and sulbactam with daptomycin against daptomycin-nonsusceptible, vancomycin-intermediate resistant *Staphylococcus aureus* (VISA) or heterogeneous vancomycin-intermediate *S. aureus* (h-VISA) isolates. The in vitro activity of daptomycin against daptomycin-nonsusceptible VISA/h-VISA isolates after adding cephalosporins with or without sulbactam was evaluated. The MIC of daptomycin against the VISA/h-VISA isolates was reduced after adding cephalosporins to daptomycin. Except for one VISA and two h-VISA isolates, the other VISA/h-VISA isolates became daptomycin-susceptible (MICs ≤ 1 mg/L). After adding sulbactam to each daptomycin/cephalosporin combination, the MIC of daptomycin against the VISA/h-VISA isolates decreased for 5 (33.3%), 6 (40.0%), 6 (40.0%), and 6 (40.0%) isolates with the cefazolin, cefmetazole, cefotaxime, and cefepime combinations, respectively. Synergism using the checkerboard method was noted in 100% of cefazolin and cefotaxime combinations and 87% and 80% of cefmetazole and cefepime combinations for all the VISA and h-VISA isolates. With the addition of sulbactam, synergism was noted in 100% of cefazolin, cefmetazole, and cefotaxime combinations and 93% of the cefepime combinations for all the VISA and h-VISA isolates. Almost all the FICs for the three-drug combinations were lower than those for the two-drug combinations. Using time-killing methods, a synergistic effect against five h-VISA isolates was observed. A synergistic effect of daptomycin, sulbactam, and each cephalosporin was observed for all VISA isolates. In conclusion, the activity of daptomycin against daptomycin-nonsusceptible VISA/h-VISA isolates can be enhanced by adding cephalosporins, and partially further promoted by sulbactam.

## 1. Introduction

Vancomycin belongs to the glycopeptide antibiotic class, and remains the drug of choice for severe methicillin-resistant *Staphylococcus aureus* (MRSA) infections [1,2]. However, an increasing number of MRSA strains have developed resistance to vancomycin, known as vancomycin-intermediate resistant *S. aureus* (VISA) or heterogeneous vancomycin-intermediate *S. aureus* (h-VISA) [3,4]. Importantly, the appropriate antibiotics for VISA/h-VISA infections, which are associated with complicated clinical courses and treatment failures, are limited [5]. Some alternative antibiotics, such as daptomycin, may be therapeutic options [5]. However, the presence of daptomycin-nonsusceptible VISA/h-VISA has been reported in recent studies. A recent study in a Brazilian teaching hospital showed that three out of six VISA isolates from bloodstream infections had a daptomycin minimal inhibitory concentration (MIC) ≥ 2 mg/L [6]. Another study demonstrated that the percentage of daptomycin-nonsusceptible isolates was 26% by E-test 15% by broth microdilution methods for h-VISA, and 62% by E-test and 38% by the broth microdilution methods for VISA [7]. Thus, combination therapy may be one way to approach the treatment of this critical condition.

Studies regarding combination therapy for VISA/h-VISA are limited. Recently, one study [8] showed that adding vancomycin to cephalosporins can enhance the antibacterial activity against VISA/h-VISA. Several studies [9,10] have also shown that β-lactam antibiotics, such as oxacillin, ceftaroline, and piperacillin-tazobactam plus vancomycin, can improve the activity of vancomycin against VISA/h-VISA. In addition, a previous study [11] demonstrated that sulbactam can enhance the activity of beta-lactam antibiotics against MRSA. Moreover, the antibacterial activity of cefazolin could be boosted by sulbactam for some *S. aureus* strains containing the ability to hyperhydrolyze it; furthermore, sulbactam also has some penicillin-binding protein (PBP)-binding activity that would complement that of cephalosporin [12]. Based on the above results and the findings of our previous study [8], we hypothesize that this additional effect of cephalosporins or sulbactam in combination with daptomycin against VISA/h-VISA may occur. In this study, we assessed the synergistic effect of a combination of cephalosporins of all generations and sulbactam with daptomycin against daptomycin-nonsusceptible VISA/h-VISA isolates. 

## 2. Materials and Methods

### 2.1. Bacterial Isolates

From 17 VISA and 38 h-VISA isolates collected from 18 hospitals (11 medical centers and 7 regional hospitals) between January 2012 and April 2014, six VISA and nine h-VISA isolates were found to be daptomycin nonsusceptible. Staphylococci were identified by colony morphology, Gram stain, and a coagulase test. The MRSA isolates were further confirmed by a tube coagulase test and growth on 6 mg/L oxacillin salt agar plates. The isolates were stored at −70 °C in Protect Bacterial Preservers (Technical Service Consultants Limited, Heywood, UK) until use. Further identification of VISA and h-VISA isolates was confirmed by population analysis profile/area under the curve ratio (PAP/AUC), as in previous reports [8,13]. Briefly, the PAP/AUC was measured for all isolates by inoculating serial 10-fold dilutions of the test organism onto increasing concentrations of vancomycin-containing brain–heart infusion (BHI) agar (Becton Dickinson, Sparks, MD, USA). Colony growth at 48 h was measured and graphed as log10 CFU/mL to obtain a PAP graph, which was used to calculate the AUC of each isolate. The ratio of the AUC of the test isolate to the AUC of *S. aureus* Mu3 (ATCC 700698) was calculated, and ratios of 0.9 to 1.3 and > 1.3 were considered h-VISA and VISA, respectively. The genetic relatedness of the isolates was examined by pulsed-field gel electrophoresis (PFGE). The daptomycin-nonsusceptible isolates were defined as isolates with a MIC of ≥2 mg/L. All of the bacterial isolates from clinical specimens were collected on a routine basis, and the analyses were carried out retrospectively. Therefore, no informed consent was required, and it was specifically waived by the Institutional Review Board. Ethics approval was obtained from the Institution Review Board of Chi Mei Medical Center (IRB: 10012-001).

### 2.2. Antibiotics and MIC Measurement

The tested antibiotics were cefazolin (CFZ), cefmetazole (CMZ), cefotaxime (CTX), cefepime (CPM), vancomycin (VA), sulbactam (Sigma, St. Louis, MO), and daptomycin (Cubist Pharmaceuticals, Lexington, MA). Except for daptomycin, all antibiotic MICs were detected by agar dilution. MIC determination by the agar dilution and microbroth dilution methods, and interpretation criteria, were based on the recommendations of the Clinical and Laboratory Standards Institute (CLSI) [14,15]. Briefly, Mueller–Hinton agar (Oxoid, Basingstoke, UK) was employed for MIC determination for *S. aureus*. Inocula were prepared by suspending growth from overnight cultures in saline to the turbidity of a 0.5 McFarland standard. Inoculated plates were then incubated in ambient air at 37 °C for 24 h. The daptomycin MIC was studied in cation-adjusted Mueller Hinton broth (CAMHB) supplemented with 50 mg/L calcium by a microbroth dilution method. In the combination MICs, the calcium concentration was added as the microbroth MIC of daptomycin. *S. aureus* ATCC 29213 was used as the control strain for each run of MIC measurements, as in a previous study [16].

### 2.3. Determination of mecA

Polymerase chain reaction (PCR) for the *mec*A gene was performed according to the protocol described by Vannuffel et al. [17]. *S. aureus* ATCC BAA-1707, USA400 was used as a positive control. All the isolates were analyzed by SCC*mec* typing and PFGE. The SCC*mec* types were determined by the multiplex PCR strategy developed by Kondo et al. [18]. DNA extraction and SmaI restriction were performed as described previously. The PFGE patterns were visually examined and interpreted according to the criteria of Tenover et al. [19]. The similarities of the PFGE profiles of each strain were compared using a Dice coefficient at 0.8% tolerance and 0.8% optimization.

### 2.4. mprF, pgsA, and cls-2 Sequencing

The *mprF* genes were amplified by PCR using previously described primers [20]. The primers for *pgsA* and *cls*-2 were designed by the NCBI Primer-Blast Tool (http://www.ncbi.nlm.nih.gov/tools/primer-blast/). The primers for *pgsA* were *pgsA*-F: 5’-GAG GAT GTA TAA TGA ATA TTC CGA ACC-3’ and *pgsA*-R: 5’-GTA TAA ACA AAT ATT TAT TTT TG-3’, and the primers for *cls*-2 were *cls*-2-F: 5’-GGT TCT CGT GGA CTG CGT AA-3’ and *cls*-2-R: 5’-ACG CCA ATT GTT CCA GA-3’. The products were sequenced in both directions by the dideoxy chain termination method in an ABI Prism 3130xl Genetic Analyzer (Applied Biosystems, Foster City, CA, USA).

### 2.5. Time-Killing Method

The inhibitory effect of the combination regimens was assessed according to the methods recommended by the CLSI [21]. In brief, the bacterial suspensions were diluted to 5 × 10^5^ colony-forming units (CFU)/mL in fresh Mueller–Hinton broth with calcium supplementation at 50 mg/L in the daptomycin-based combination test [22]. The susceptible breakpoint concentrations were used for daptomycin and the four cephalosporins, i.e., 1, 8, 16, 8, and 8 mg/L, respectively, and sulbactam was adjusted to 8 mg/L according CLSI guidelines for *Staphylococcus* spp [15,23]. The bacterial counts were measured at 0, 4, 8, and 24 h by enumerating the colonies in 10-fold serially-diluted specimens of 100 µL aliquots plated on Mueller–Hinton agar (Difco Laboratories, Sparks, MD) at 37 °C [24]. In this study, daptomycin was combined with a cephalosporin with or without sulbactam. All experiments were performed in duplicate. Synergism was defined as a ≥2 log10 decrease in CFU/mL between the combination regimen and its most active constituent after 24 h, as well as the number of surviving organisms in the combination regimen, which had to be ≥2 log10 CFU/mL below the starting inoculum. Bacteriostatic and bactericidal activities were defined as <3 log10 and ≥3 log10 reductions, respectively, in CFU/mL at 24 h relative to the starting inoculum. In addition, at least one of the combinations of drugs had to be present at a concentration that did not affect the growth of the test organism.

### 2.6. Daptomycin MIC Change

The MICs of daptomycin combined with a cephalosporin, or daptomycin combined with a cephalosporin and sulbactam, were determined by the microbroth dilution method as described above, modified from the CLSI’s recommendations [14,15]. The medium pre-addition susceptible breakpoint concentration of cephalosporin or susceptible breakpoint concentration of cephalosporin with 8 mg/L sulbactam was used in the combination test. All MIC levels were tested three times, and the mean of the three MICs was recorded.

### 2.7. Checkerboard Method

The microdilution checkerboard method was used to calculate the fractional inhibitory concentrations (FICs) for daptomycin combined with a cephalosporin, which was modified according to the CLSI [8,14,15]. In the three-drug combination checkerboard, daptomycin combined with a cephalosporin was also combined with 8 mg/L sulbactam in each well. The following formula was used to calculate the FIC index: the FIC of daptomycin (MIC of daptomycin in combination/MIC of daptomycin alone) + the FIC of the cephalosporin (MIC of cephalosporin in combination/MIC of cephalosporin alone). The definition of FIC_90_ is the 90% fractional inhibitory concentration (FIC_90_) of clinical isolates against a combination of test drugs. Synergism was defined as an FIC index of ≤0.5, a no-interaction FIC index was >0.5 but ≤4, and an antagonism FIC index >4 [25].

## 3. Results

### 3.1. The Results of the MIC Tests

Table 1 shows the MICs of cephalosporin-group antimicrobials, vancomycin, sulbactam, and daptomycin against the six VISA and nine h-VISA isolates. The MIC ranges of vancomycin against VISA and h-VISA isolates were 4 mg/L and 1–2 mg/L, respectively. All the VISA and h-VISA isolates were resistant to every cephalosporin, based on the MIC level. The MICs of daptomycin against VISA and h-VISA isolates were all 2 mg/L.

### 3.2. Molecular Characteristics

Based on the PFGE analysis, the VISA and h-VISA isolates could be classified into eleven different patterns. In addition to one h-VISA that was nontypeable, three VISA isolates belonged to SCC*mec* II and III. Six h-VISA isolates belonged to SCC*mec*III, and the other two isolates belonged to SCC*mec* II and V. Three VISA and seven h-VISA isolates had a *pgsA* mutation. Three h-VISA isolates had a *cls*-2 mutation. All the VISA and h-VISA isolates had *mprF* mutations (Supplementary Materials Table S1).

### 3.3. Changes in MIC Levels

The daptomycin MICs for the VISA/h-VISA isolates with and without a cephalosporin-susceptible breakpoint concentration are shown in Table 2. The MICs of daptomycin against VISA/h-VISA isolates were lower when combined with cephalosporins than when daptomycin alone was applied. CMZ or CFZ resulted in the lowest daptomycin MICs against VISA/h-VISA isolates. Except for one VISA (V18) and two h-VISA (HV62 and HV355) isolates, all the other VISA/h-VISA isolates became daptomycin susceptible, with MICs ≤ 1 mg/L. After adding sulbactam to each daptomycin/cephalosporin combination, the MIC of daptomycin against VISA/h-VISA decreased for 5 (33.3%), 6 (40.0%), 6 (40.0%), and 6 (40.0%) isolates with the CFZ, CMZ, CTX, and CPM combinations, respectively.

### 3.4. Checkerboard Method

With the checkerboard method, the FIC_90_ of the daptomycin-based combinations ranged from 0.133 to 0.5 for the VISA isolates (Table 3). One isolate, V25, with a relatively high FIC had an FIC_90_ of approximately 0.625–0.75 when combined with CMZ and CPM. Likewise, in the h-VISA isolates, the FIC_90_ of the daptomycin-based combinations ranged from 0.127 to 0.5, and only three isolates (HV44, HV62, and HV355) had relatively high FICs of approximately 0.516–0.563 when CMZ and CPM were combined. Synergism was noted in 100% of the CFZ and CTX combinations and 87% and 80% of the CMZ and CPM cephalosporin combinations, respectively, for all of the VISA and h-VISA isolates. No antagonism was observed for any of the combinations.

With the further addition of sulbactam, the FIC_90_ of the daptomycin-based combinations ranged from 0.064 to 0.313 for all the VISA and h-VISA isolates (Table 3). Only one isolate, V25, had a relatively high FIC of 0.563 when combined with CPM. Synergism was noted in 100% of the CFZ, CMZ, and CTX combinations and 93% of the 4^th^ cephalosporin combination for all the VISA and h-VISA isolates. Overall, almost all the FICs of the three drug combinations (plus sulbactam) were lower than those of the two drugs alone. Additionally, no antagonism was observed for any of the three-drug combinations.

### 3.5. Time-killing Methods

Time-killing assays for each isolate are shown in Table 4. For almost all the VISA isolates, either bactericidal or bacteriostatic effects were observed with the combinations of cephalosporins. For only one strain, V23, we did not find synergistic effects with the combination of daptomycin and CFZ. For h-VISA isolates, a synergistic effect was observed against five of the nine h-VISA isolates (HV9, HV44, HV62, HV74, and HV85) under the combination of daptomycin and each cephalosporin, namely, CFZ, CMZ, CTX, and CPM. In contrast, an insignificant effect was observed for the other four h-VISA isolates (HV4, HV83, HV204, and HV355) with a combination of daptomycin and each of the cephalosporins.

We assessed the synergistic effect of daptomycin, sulbactam, and each cephalosporin; synergistic effects were observed for all of the VISA isolates. Furthermore, according to colony counts, the synergistic effects were more significant with all three drugs than with a combination of daptomycin and cephalosporin only. For h-VISA, no synergistic effect was found in combination with CFZ for two isolates (HV83 and HV355), in combination with CMZ for two isolates (HV4 and HV355), in combination with CTX for three isolates (HV4, HV204, HV355), or in combination with CPM for three isolates (HV4, HV204, and HV355). For the other h-VISA isolates, synergistic effects were observed for various combinations. According to colony counts, the synergistic effects against h-VISA with all three drug types were more significant than with the combination of daptomycin and cephalosporin alone. With these three drug combinations including sulbactam, the bactericidal effect seems to be more pronounced than that with two-drug combinations. We did not find any bacteriostatic or bactericidal synergistic effects against the VISA or h-VISA isolates with the combination of daptomycin and sulbactam. Overall, the bactericidal effect seems to be more predominant when comparing the two- and three-drug combinations in every group, namely, CFZ (from 6 to 11 isolates), CMZ (from 7 to 11 isolates), CTX (from 7 to 8 isolates), and CPM (from 8 to 10 isolates).

## 4. Discussion

This study assessed the effect of the addition of different cephalosporins and sulbactam with daptomycin against VISA/h-VISA isolates and produced several significant findings. First, we demonstrated the synergistic effect of daptomycin and different cephalosporins against VISA/h-VISA isolates with different methods, including an MIC test, a checkerboard assay, and a time-killing method. A previous study [26] showed that the combination of ceftaroline and daptomycin (6 or 10 mg/kg/day) can produce a >5 log10 CFU/mL reduction within 96 h against MRSA isolates. In a clinical trial, daptomycin plus ceftaroline hastened the clearance of 26 cases of refractory staphylococcal bacteremia, which included two VISA cases [27]. Instead of ceftaroline, a fifth-generation cephalosporin, we used the first- to fourth-generation cephalosporins for investigation. Using the time-killing method, for almost all the VISA isolates, either bactericidal or bacteriostatic effects were observed with the combinations of cephalosporins. Regarding h-VISA isolates, a synergistic effect was observed against five of the nine h-VISA isolates under the combination of daptomycin and each cephalosporin. Our findings were consistent with previous in vitro study [10], the combination of vancomycin plus ceftaroline showed synergy against 5 of 5 VISA and 4 of 5 hVISA using the time-killing method tested. Furthermore, we demonstrated that the additional effects of these cephalosporins with daptomycin can help reduce the MICs of daptomycin against daptomycin-nonsusceptible VISA/h-VISA. The synergy between cephalosporins and daptomycin may be explained by the “seesaw effect”, in which beta-lactams thin the cell wall to allow vancomycin to bind to target sites during cell wall synthesis, or in which beta-lactams increase the negative cell surface charge to allow improved daptomycin binding and bactericidal activity to occur [28]. In addition, the synergistic activity may involve differential PBP binding, which was suggested since beta-lactams with PBP1 activity, such as cefazolin and meropenem, seem to possess the best synergistic activity with daptomycin against *S. aureus* [29,30]. Based on these findings, cephalosporins and daptomycin may be a potent combination against VISA/h-VISA. Clinically, the synergy of antibiotic combinations may accelerate pathogen clearance in patients with high bacterial loads, and broaden the antimicrobial spectrum to decrease the risk of initial inappropriate treatment. However, in vitro activity does not equal in vivo response. Further well-designed prospective studies are needed to provide more convincing evidence to fully assess the value of the effects of these newer clinical combinations.

Second, we added sulbactam to the combination of cephalosporins and daptomycin to determine whether there were additional effects against VISA/h-VISA. Initially, the MICs of sulbactam for VISA/h-VISA remained very high, and no synergistic effect of sulbactam and daptomycin against VISA/h-VISA was noted. However, among several isolates when sulbactam was added into the combination of cephalosporins and daptomycin, the MIC level of daptomycin that was effective against VISA/h-VISA was reduced by cephalosporin/daptomycin/sulbactam combinations compared to that of the two antibiotic combinations of cephalosporins and daptomycin alone. The time-killing methods also showed an additional synergistic effect with the addition of sulbactam to the combination of cephalosporin and daptomycin. Although previous studies [31,32,33] have shown a synergistic effect of sulbactam in various combinations, such as arbekacin with ampicillin-sulbactam, ours is the first to report a synergistic effect of daptomycin and cephalosporins with sulbactam against daptomycin-nonsusceptible VISA/h-VISA.

Third, the MICs of cephalosporins for VISA/h-VISA remain very high, as seen in our previous study [8]. In this study, we used susceptible breakpoint concentrations of cephalosporins that were far lower than the MIC, and still found an enhanced synergistic effect of cephalosporins/sulbactam and daptomycin, as evidenced by a significant reduction in the daptomycin MIC. These findings indicated that even though VISA/h-VISA is resistant to cephalosporins alone, combinations of antimicrobials and very low concentrations of cephalosporins can play useful roles in daptomycin therapy for daptomycin-nonsusceptible VISA/h-VISA.

Finally, among these daptomycin-nonsusceptible VISA/h-VISA isolates, all had various mutations for the development of reduced susceptibility to daptomycin. All the VISA and h-VISA isolates had *mprF* mutations. Three of the VISA isolates and seven of the h-VISA isolates had a *pgsA* mutation. Three h-VISA isolates had a *cls*-2 mutation. Moreover, some strains had two or three of these mutations simultaneously. The mechanisms of these mutations have been reported in a previous study [34], and mutations in genes encoding membrane phospholipid biosynthesis may reduce the net negative charge of the cell membrane in *S. aureus* strains [35].

This study had several limitations. First, the number of clinical isolates -VISA/h-VISA was limited. Second, we do not have fifth generation ceftaroline to assess its combination effect with daptomycin. Further large-scale study is needed.

## 5. Conclusions

This report is the first in vitro study to use different methods to evaluate the synergistic effects of cephalosporin, sulbactam, and daptomycin combinations. The in vitro activity of daptomycin against daptomycin-nonsusceptible VISA/h-VISA isolates can be enhanced by adding cephalosporins and, occasionally, further promoted by sulbactam. 

## Figures and Tables

**Table 1:** Minimal inhibitory concentrations (MICs) (mg/L) of eight drugs against six VISA and nine h-VISA isolates.

Antibiotics	VISA						h-VISA								
	V18	V19	V23	V25	V26	V31	HV4	HV9	HV44	HV62	HV74	HV83	HV85	HV204	HV355
CFZ	128	128	128	128	64	128	128	128	256	512	256	256	256	512	256
CMZ	128	128	64	64	64	64	64	128	64	256	64	64	64	128	128
CTX	1024	1024	1024	1024	1024	512	512	512	1024	>1024	512	512	512	>1024	1024
CPM	128	128	128	128	128	128	64	128	512	512	512	256	512	512	512
VA	4	4	4	4	4	4	2	2	2	2	1	2	2	2	2
SUL	>1024	>1024	>1024	>1024	>1024	>1024	>1024	>1024	>1024	>1024	>1024	>1024	>1024	>1024	>1024
DAP	2	2	2	2	2	2	2	2	2	2	2	2	2	2	2

Note. CFZ = cefazolin, CMZ = cefmetazole, CTX = cefotaxime, CPM = cefepime, VA = vancomycin, SUL = sulbactam, DAP = daptomycin.

**Table 2:** The MICs of daptomycin/cephalosporin combinations with or without sulbactam for VISA and h-VISA isolates.

Isolates	DAP + CFZ		DAP + CMZ		DAP + CTX		DAP + CPM	
	-	+SUL	-	+SUL	-	+SUL	-	+SUL
V18	1	0.5	1	0.83	1	1	1.33	0.67
V19	0.5	0.5	0.5	0.5	1	0.5	0.5	0.5
V23	0.5	0.5	0.5	0.5	1	0.83	0.83	0.5
V25	0.5	0.5	0.5	0.5	0.5	0.5	0.5	0.5
V26	1	0.5	0.5	0.25	1	1	1	0.5
V31	0.5	0.5	0.25	0.25	0.5	0.5	0.5	0.5
HV85	0.25	0.25	0.25	0.21	0.25	0.25	0.5	0.42
HV74	0.5	0.25	0.25	0.25	0.5	0.25	0.5	0.5
HV83	0.25	0.25	0.25	0.25	0.25	0.25	0.25	0.25
HV4	0.5	0.25	0.5	0.25	0.5	0.25	0.5	0.42
HV9	0.42	0.25	0.25	0.25	0.25	0.25	0.25	0.25
HV44	1	0.5	1	0.5	1	0.5	1	1
HV62	1	1	1	1	1.67	1	1	1
HV204	1	1	1	1	1	1	1	1
HV355	1	1	1	0.83	1	1	1.67	1
Mean ± SD	0.66 ± 0.30	0.52 ± 0.27	0.58 ± 0.32	0.49 ± 0.29	0.76 ± 0.40	0.61 ± 0.33	0.76 ± 0.41	0.60 ± 0.27

DAP: 1 mg/mL, CFZ: 8 mg/mL, CMZ: 16 mg/mL, CTX: 8 mg/mL, CPM: 8 mg/mL, SUL: 8 mg/mL. SD = standard deviation; daptomycin = DAP; cefazolin = CFZ; cefmetazole = CMZ; cefotaxime = CTX; cefepime = CPM; sulbactam = SUL.

**Table 3:** The FIC results of the checkerboard method of daptomycin/cephalosporin combinations with or without sulbactam against VISA and h-VISA isolates.

Isolates	DAP + CFZ		DAP + CMZ		DAP + CTX		DAP + CPM	
	-	+ SUL	-	+ SUL	-	+ SUL	-	+ SUL
**V18**	0.313	0.126	0.500	0.254	0.266	0.254	0.156	0.126
**V19**	0.250	0.129	0.375	0.250	0.258	0.250	0.375	0.252
**V23**	0.281	0.252	0.500	0.184	0.281	0.251	0.281	0.125
**V25**	0.313	0.252	0.750	0.250	0.250	0.250	0.625	0.563
**V26**	0.133	0.127	0.281	0.141	0.156	0.126	0.133	0.127
**V31**	0.252	0.129	0.313	0.188	0.250	0.251	0.258	0.252
**HV4**	0.141	0.129	0.188	0.141	0.156	0.127	0.252	0.252
**HV9**	0.129	0.125	0.141	0.133	0.251	0.251	0.250	0.251
**HV44**	0.258	0.254	0.516	0.313	0.375	0.188	0.500	0.188
**HV62**	0.258	0.252	0.281	0.254	0.266	0.254	0.531	0.252
**HV74**	0.141	0.127	0.141	0.133	0.250	0.126	0.127	0.127
**HV83**	0.141	0.129	0.281	0.250	0.127	0.064	0.129	0.127
**HV85**	0.188	0.133	0.141	0.141	0.127	0.126	0.133	0.127
**HV204**	0.266	0.252	0.266	0.258	0.254	0.252	0.266	0.266
**HV355**	0.266	0.251	0.254	0.254	0.500	0.251	0.563	0.252
**Range**	0.129~0.313	0.126~0.251	0.141~0.750	0.133~0.313	0.127~0.500	0.064~0.254	0.127~0.625	0.125~0.563
**Mean ± SD**	0.222 ± 0.068	0.178 ± 0.063	0.329 ± 0.172	0.210 ± 0.060	0.251 ± 0.096	0.201 ± 0.068	0.305 ± 0.173	0.219 ± 0.113

DAP: 1 mg/mL, CFZ: 8 mg/mL, CMZ: 16 mg/mL, CTX: 8 mg/mL, CPM: 8 mg/mL, SUL: 8 mg/mL. SD = standard deviation; daptomycin = DAP; cefazolin = CFZ; cefmetazole = CMZ; cefotaxime = CTX; cefepime = CPM; sulbactam = SUL.

**Table 4:** The log change (log10 CFU/mL) from the starting inoculum and the most active single agent after 24 h of incubation with susceptible breakpoint concentrations of daptomycin/cephalosporin combinations with or without sulbactam against VISA and h-VISA isolates.

Changes in Colony Count in Response to the Most Active Single Drug, log_10_ CFU/mL									
Isolates	DAP+ CFZ	DAP+ CFZ	DAP+ CMZ	DAP+ CFZ	DAP+ CTX	DAP+ CTX	DAP+ CPM	DAP+ CPM	DAP+ SUL
		+SUL		+SUL		+SUL		+SUL	
**V18**	−2.70^a^	−4.34^b^	−2.79^a^	−4.34^b^	−3.14^b^	−2.81^a^	−2.84^a^	−2.79^a^	0.00
**V19**	−2.49^a^	−3.90^b^	−3.90^b^	−3.90^b^	−2.24^a^	−2.30^a^	−2.60^a^	−3.90^b^	−1.40
**V23**	−1.55	−2.92^a^	−2.70^a^	−4.00^b^	−2.80^a^	−2.70^a^	−3.40^b^	−4.00^b^	−1.42
**V25**	−4.53^b^	−4.53^b^	−4.53^b^	−4.53^b^	−4.53^b^	−4.53^b^	−4.53^b^	−4.53^b^	−2.00
**V26**	−2.09^a^	−2.49^a^	−2.41^a^	−2.45^a^	−2.41^a^	−2.45^a^	−2.41^a^	−2.45^a^	0.33
**V31**	−2.85^a^	−4.32^b^	−2.90^a^	−2.90^a^	−2.90^a^	−3.76^b^	−3.85^b^	−3.92^b^	−1.38
**HV4**	−4.07	−5.10^b^	−3.36	−3.74	−3.62	−4.06	−2.10	−3.10	0.00
**HV9**	−4.52^b^	−5.66^b^	−5.40^b^	−5.42^b^	−5.70^b^	−6.22^b^	−5.14^b^	−7.00^b^	−0.24
**HV44**	−6.00^b^	−5.80^b^	−7.00^b^	−6.40^b^	−5.92^b^	−7.00^b^	−6.10^b^	−6.70^b^	−0.63
**HV62**	−5.12^b^	−4.42^b^	−7.00^b^	−5.38^b^	−7.00^b^	−5.38^b^	−5.80^b^	−5.80^b^	−1.52
**HV74**	−4.59^b^	−6.10^b^	−5.85^b^	−6.00^b^	−4.70^b^	−6.70^b^	−4.52^b^	−6.22^b^	−1.25
**HV83**	−3.40	−4.25	−4.27	−5.30^b^	−4.59	−5.47^b^	−4.00	−5.15^b^	0.00
**HV85**	−4.17^b^	−5.70^b^	−6.10^b^	−7.00^b^	−6.22^b^	−7.00^b^	−5.85^b^	−7.00^b^	−0.20
**HV204**	−4.55	−5.18^b^	−5.06	−4.70^b^	−2.52	−3.27	−4.18	−4.22	−0.02
**HV355**	−2.47	−3.25	−3.40	−4.38	−1.22	−2.80	−0.49	−3.22	−2.00
**Mean**	−3.67	−4.53	−4.44	−4.70	−3.97	−4.43	−3.85	−4.67	−0.78
**SD**	1.27	1.08	1.56	1.25	1.70	1.74	1.58	1.55	0.81
**Bacteriostatic**	4	2	4	2	4	4	3	2	0
**Bactericidal**	6	11	7	11	7	8	8	10	0

DAP: 1 mg/mL, CFZ: 8 mg/mL, CMZ: 16 mg/mL, CTX: 8 mg/mL, CPM: 8 mg/mL, SUL: 8 mg/mL Synergism was defined as at least a 100-fold reduction in bacterial load between the combination and the most active constituent after 24 h. ^a^ Bacteriostatic with synergistic effect: the presence of a ≥ 2 log10 but < 3 log10 reduction in CFU/mL at 24 h relative to the initial inoculum. ^b^ Bactericidal with synergistic effect: the presence of a ≥ 3 log10 reduction in CFU/mL at 24 h relative to the initial inoculum. Data at the 4th hour are not shown

## Supplementary Materials

The following are available online at https://www.mdpi.com/2079-6382/8/4/184/s1, Table S1: PFGE, SCCmec type and resistance genes of six VISA and nine h-VISA isolates.
