# Supplementary material for: The Potential Role of Sulbactam and Cephalosporins Plus Daptomycin Against Daptomycin-Nonsusceptible VISA and H-VISA Isolates: An In Vitro Study
Source: Antibiotics (Basel). 2019 Oct 14;8(4):184. doi: 10.3390/antibiotics8040184 (PMC6963809; doi:10.3390/antibiotics8040184)
Supplement: Supplementary file 1 [file antibiotics-08-00184-s001.pdf]

antibiotics MDPI

Supplement Table 1. PFGE, SCCmec type and resistance genes of six VISA and nine h-VISA isolates.

Isolates	PFGE	SCC*mec*	*pgsA*		*cls-2*		*mprF*	
			Nucleotide	Protein	Nucleotide	Protein	Nucleotide	Protein
**V18**	G1	II	1 site SM (378 C→ T)	WT	Multiple site (7) SMs	WT	Multiple site (13) SMs 77 T →C	V26A
V19	G2	II	1 site SM (378 C→ T)	WT	Multiple site (7) SMs	WT	Multiple site (13) SMs 77 T →C	V26A
V23	C1	III	475 G→A	G159S	Multiple site (7) SMs	WT	Multiple site (11) SMs 77 T →C, 2074 C→ G	V26A, Q692E
V25	C1	III	475 G→A	G159S	Multiple site (7) SMs	WT	Multiple site (11) SMs 77 T →C, 2074 C→ G	V26A, Q692E
V26	G3	II	1 site SM	WT	Multiple site (7) SMs	WT	Multiple site (13) SMs 77 T →C	V26A
V31	B	III	475 G→A	G159S	Multiple site (7) SMs	WT	Multiple site (11) SMs 77 T →C, 2074 C→ G	V26A, Q692E
HV4	E	III	475 G→A	G159S	779 deletion A	Truncates protein to 277 aa	Multiple site (11) SMs 77 T →C, 2074 C→ G	V26A, Q692E
HV9	D2	III	475 G→A	G159S	Multiple site (7) SMs	WT	Multiple site (11) SMs 77 T →C, 2074 C→ G	V26A, Q692E
HV44	A	V	475 G→A	G159S	Multiple site (7) SMs 787 T→A	F263I	Multiple site (11) SMs 77 T →C, 1034 C→A, 2074 C→ G	V26A, T345I, Q692E
HV62	H1	II	1 site SM (378 C→ T)	WT	Multiple site (7) SMs	WT	Multiple site (13) SMs 77 T →C, 1034 C→A	V26A, T345K
HV74	F1	III	475 G→A	G159S	Multiple site (7) SMs	WT	Multiple site (11) SMs 77 T →C, 2074 C→ G	V26A, Q692E
HV83	F2	III	475 G→A	G159S	Multiple site (7) SMs	WT	Multiple site (11) SMs 77 T →C, 2074 C→ G	V26A, Q692E
HV85	C2	III	475 G→A	G159S	Multiple site (7) SMs 1064 A→C	D355A	Multiple site (11) SMs 77 T →C, 2074 C→ G	V26A, Q692E
HV204	H2	ND	1 site SM (378 C→ T)	WT	Multiple site (7) SMs	WT	Multiple site (13) SMs 77 T →C, 1033 A→G	V26A, T345A
HV355	D1	III	475 G→A	G159S	Multiple site (7) SMs	WT	Multiple site (11) SMs 77 T →C, 2074 C→ G, 2476 C →T	V26A, Q692E, L826F

SM = silent mutation; WT = wild type
